# Draft Genome Sequences of Two Burkholderia multivorans Sequential Isolates from a Chronic Lung Infection of a Cystic Fibrosis Patient

**DOI:** 10.1128/genomeA.01531-14

**Published:** 2015-02-12

**Authors:** Inês N. Silva, Pedro M. Santos, Leonilde M. Moreira

**Affiliations:** a Institute for Bioengineering and Biosciences (iBB), Instituto Superior Técnico, Universidade de Lisboa, Lisbon, Portugal; b Department of Biology, Centro de Biologia Molecular e Ambiental (CBMA), Universidade do Minho, Campus de Gualtar, Braga, Portugal; c Department of Bioengineering, Instituto Superior Técnico, Universidade de Lisboa, Lisbon, Portugal

## Abstract

Burkholderia multivorans belongs to the Burkholderia cepacia complex, which comprises opportunistic pathogens infecting cystic fibrosis (CF) patients. Here, we report the genome sequences and annotations of two sequential B. multivorans clinical isolates (D2095 and D2214) displaying different traits. The differences in the genomic contents of these isolates may provide clues regarding the evolution of B. multivorans within the airways of a CF patient.

## GENOME ANNOUNCEMENT

The Burkholderia cepacia complex comprises 18 species of closely related betaproteobacteria capable of establishing infections in cystic fibrosis (CF) and immunocompromised patients (1–3). These microorganisms have large genomes and a high gene content, providing them with the ability to rapidly adapt and colonize different niches, like the airways of CF patients (4). Within the B. cepacia complex, the species Burkholderia multivorans is one of the most frequently isolated from CF patients (2). Moreover, B. multivorans undergoes phenotypic changes while chronically colonizing CF airways, and mucoid-to-nonmucoid transitions within CF lungs have been reported (5, 6). A genomic comparison between two B. multivorans sequential isolates that underwent a mucoid-to-nonmucoid morphotype transition within the CF lung is lacking. Here, we report the genome sequences of two isolates, D2095 and D2214, isolated from the same CF patient attending a Vancouver CF clinic. D2095 was isolated from a throat swab in June 2006 and was highly mucoid in yeast-extract-mannitol (YEM) solid medium due to exopolysaccharide production, while D2214 was isolated from a sputum sample in November 2006 and displayed a nonmucoid morphotype (5). An extensive phenotypic characterization of these isolates, together with transcriptomic profiling and morphotype stability studies, was performed (7, 8).

Genomic DNA from B. multivorans D2095 and D2214 was extracted and purified using the DNeasy blood and tissue kit (Qiagen), according to the manufacturer’s instructions. Genomic libraries were prepared using the TruSeq SBS kit version 5 (Illumina), and genome sequencing was performed using Illumina HiSeq 2000 technology, giving rise to paired-end libraries of 2 × 100 bp, with an insert size of approximately 300 bp (~1,000× coverage). The raw paired-end reads of D2095 and D2214 were assembled using Edena version 3.131028 (9), and the assembly of the genomes was finished using SSPACE Premium version 2.3 (10). For D2095 scaffolding, an additional paired-end library and a mate-pair library were used. Genomic DNA of B. multivorans D2095 was resequenced using Illumina HiSeq 2000 technology, yielding an extra 2 × 100-bp paired-end library, with an insert size of 300 bp (~80× coverage), and a 2 × 50 mate-pair library, with an insert size of 5 kb (~70× coverage). The D2095 and D2214 genomes were assembled into 15 and 26 contigs, accounting for 6,668,882 bp and 6,465,997 bp and estimated G+C contents of 67.1% and 67.4%, respectively. The D2214 assembly was more fragmented due to the nonusage of a mate-pair library during the scaffolding process. Both genome drafts were annotated using the NCBI Prokaryotic Genomes Automatic Annotation Pipeline, which revealed that the D2095 isolate possesses 5,952 open reading frames (ORFs), whereas D2214 has 5,782 ORFs. A major genome deletion of 175 kb was identified in D2214, consisting mainly of genes encoding hypothetical proteins. We also identified in the D2214 genome a 37-kb genome duplication of phage-related protein-coding genes. Further comparative genomics of both isolates will bring insights into the mechanisms of virulence/persistence and evolution employed by B. multivorans within the airways of a CF patient.

### Nucleotide sequence accession numbers. 

The B. multivorans D2095 and D2214 whole-genome shotgun sequence projects were deposited in DDBJ/EMBL/GenBank under the accession numbers JFHP00000000 (D2095) and JFHQ00000000 (D2214). The versions described in this paper are the first versions.
